# Peer review of "Biphenarenes, Versatile Synthetic Macrocycles for Supramolecular Chemistry"

_molecules, 2023, doi:10.3390/molecules28114422_

Round 1

Reviewer 1 Report

This review by Zhang et al. describes potential uses of a family of cyclophanes termed biphenarenes, which was recently introduced by Li and co-workers, in areas ranging from adsorption and separation to sensing. Most of the investigations were apparently performed by groups from China as demonstrated by the somewhat small number of references referring to the work of groups from other countries. However, the properties biphenarenes are clearly interesting and it is therefore warranted to introduce them in a review to a wider audience. Acceptance of this manuscript can therefore be recommended once the following issues have been addressed.

Considering that the review intends to introduce a structurally versatile family of macrocycles, it is surprising (and somewhat disappointing) how little information is provided about what biphenarenes actually are. The authors should clearly explain in Section 2 the structural parameters that qualify a macrocycle to be called biphenarene. In addition, they must also include information about the possible synthetic approaches to prepare biphenarenes. Is it necessary to adapt the synthetic approach when using different building blocks as subunits? How is ring size controlled? Importantly, it would also be important to refer to the scale at which the syntheses can be performed and the amount of product that can thus be obtained? How is purification be achieved? The potential applicability of biphenarenes hinges on the ease of preparation and the amount of product that is accessible.

The authors also repeatedly use the somewhat qualitative term "large macrocycles". They should specify typical diameters of biphenarenes and compare them with the cavity diameters of other macrocyclic hosts.

Finally, since the authors repeatedly refer to the effects of ring size on the conformational behavior of traditional supramolecular hosts, they must clearly explain the conformational behavior of biphenarens. What is known about the conformations of these compounds and how does conformation depend on ring size. Readers interested in using biphenarenes for their purposes must know more about these aspects, without which it is impossible to decide whether biphenarenes are alternatives to other macrocyclic hosts in certain areas.

Another aspect that can be improved relates to the figures. At the moment, the authors almost only use figures from previous publications. These figures were probably useful in the corresponding publication to illustrate the reported results, but they are rather confusing outside of the original contexts. Figure 4 is an especially problematic example. Is it really necessary, for example, to include the NOESY NMR spectrum? Importantly, since the structures of biphenarenes investigated in the cited publications are all drawn differently, it is very difficult for the reader to easily understand the structural relationship of the compounds.

In addition to these general aspects, a number of linguistic aspects should be considered to improve the readability of the manuscript.

- The authors consistently use the term "supramolecular macrocycle(s)", which is not well chosen. The macrocycles to which they refer are molecules prepared by typical concepts of molecular chemistry, and it is only their ability to act as receptors that qualifies them for supramolecular chemistry. The term "supramolecular" refers to properties or concepts but cannot be used to characterize covalently assembled molecules. This reviewer therefore strongly suggests to replace the term "supramolecular macrocycles" wherever it appears in the manuscript with terms such as "supramolecular hosts", "macrocyclic hosts", "macrocyclic receptors", "cyclophane-based hosts" etc.

- In the first sentence the authors write "...laid the foundation for the development of non-covalent interactions between molecules" which is awkward and wrong. Non-covalent interactions between molecules have always existed and they did not have to be developed. Pedersen's discovery "laid the foundation for the development of synthetic molecules that could engage in non-covalent interactions."

- Line 39: "the selective complexation of biphenarenes with cationic, anionic and neutral guest molecules" must read "the selective complexation of cationic, anionic and neutral guest molecules with biphenarenes."

- Line 61: "guest or molecules" should read "guest molecules" (a guest is usually also a molecule).

- Line 75: In the sentence "prone to deformation and folding due to the directivity of covalent bonds" the second part "due to the directivity (directionality is probably the better term) of covalent bonds" can be omitted. Importantly, this argument should also hold for biphenarenss. The authors must explain why "deformation and folding" is apparently irrelevant for these cyclophanes. It is also unclear what the word "it" in the next sentence refers to.

- Line 87: See comment referring to line 61.

- Line 115: Is it really correct to state that "Molecular recognition plays an important role in biological system[s], ion detection and environmental pollution control" only? Molecular recognition is relevant in so many areas that it is incorrect to name only three.

- Line 118: Remove the word "topological", which – in the context of this sentence – has the same meaning as structure. "Topological structure" is therefore a pleonasm.

- Line 134: "protons of G2 exhibited field displacement" should read "protons of G2 were shielded."

- Line 163: The sentence "This is beneficial for applications in sensors, nanomaterials, ion or molecular transport, and supramolecular amphiphiles" could be improved by stating "This renders them useful as sensors, nanomaterials, ion or molecular transporters, and supramolecular amphiphiles."

- Line 171: "They can efficiently extract..." should read "They can be used to efficiently extract..."

- Line 212: The phrase "stably present in the cavity" indicates that benzene and toluene are unstable outside the cavity, which is wrong. Therefore "stably present" should probably better read "efficiently bound."

- Line 221: What is a "cyclic experiment"? Would it be better to state "Performing the experiment repeatedly"?

- Line 250: Again "cyclic adsorption properties" is not a well-chosen term. The "properties" are not "cyclic". Please rephrase.

- Line 313: The word "reach" in this sentence is likely incorrect. Would it be correct to say "these results indicate"?

- Line 370: This paragraph only repeats what has been written before. It can completely be removed except for the final sentence stating "Overall, the fascinating..."

See my comments above.

Author Response

Reply to reviewer 1:

  1. This review by Zhang et al. describes potential uses of a family of cyclophanes termed biphenarenes, which was recently introduced by Li and co-workers, in areas ranging from adsorption and separation to sensing. Most of the investigations were apparently performed by groups from China as demonstrated by the somewhat small number of references referring to the work of groups from other countries. However, the properties biphenarenes are clearly interesting and it is therefore warranted to introduce them in a review to a wider audience. Acceptance of this manuscript can therefore be recommended once the following issues have been addressed. Considering that the review intends to introduce a structurally versatile family of macrocycles, it is surprising (and somewhat disappointing) how little information is provided about what biphenarenes actually are. The authors should clearly explain in Section 2 the structural parameters that qualify a macrocycle to be called biphenarene. In addition, they must also include information about the possible synthetic approaches to prepare biphenarenes. Is it necessary to adapt the synthetic approach when using different building blocks as subunits? How is ring size controlled? Importantly, it would also be important to refer to the scale at which the syntheses can be performed and the amount of product that can thus be obtained? How is purification be achieved? The potential applicability of biphenarenes hinges on the ease of preparation and the amount of product that is accessible.

Many thank you for your kind advices and comments. These were all done. Biphen[n]arenes, including basic biphen[n]arenes, functional biphen[n]arenes, and cage compounds, are new macrocyclic hosts appeared in the supramolecular world recently. Typically, biphenarenes are made up of 4,4’-biphenol or 4,4’-biphenol ether units linked by methylene bridges at the 3- and 3’- positions. The synthesis of biphenarenes is based on the linking of reaction modules to form macrocycles by Friedel-Crafts alkylation. In addition, modular synthetic strategy is a versatile method for the synthesis of biphenarenes, which can increase the cavity sizes by changing the structural units. Biphenarenes with different structural units can be constructed by these two synthesis methods. For example, the cavity sizes of biphenarenes can be easily increased using long and rigid structural units or increasing the number of structural units. Meanwhile, gram-scale synthesis of biphenarenes is easily achieved in the laboratory. The purification of biphenarenes can be achieved by column chromatography and recrystallization. Furthermore, biphenarenes are easy to prepare since they can be obtained by one-step condensation reaction using commercial reagents. Biphenarenes show good performance in adsorptive separation, sensing and drug delivery, and have broad application prospects in chemistry, biology, materials science and other fields.

  1. The authors also repeatedly use the somewhat qualitative term "large macrocycles". They should specify typical diameters of biphenarenes and compare them with the cavity diameters of other macrocyclic hosts.

Thank you very much for your kind advices. This was done. The comparison results are shown in Table 1 and Table 2.

Table 1. Chemical structures and diameters of traditional macrocyclic hosts.

Macrocyclic host

Chemical structure

Diameter (Å)

Ref.

α-Cyclodextrin

4.7-5.3

J. Am. Chem. Soc. 2021, 143, 1984–1992.

β-Cyclodextrin

6.0-6.5

Nat. Commun. 2023, 14, 1284.

γ-Cyclodextrin

7.5-8.3

Chem 2021, 7, 2190–2200.

Per-ethylated pillar[5]arene

4.7

Chem. Rev. 2016, 116, 7937–8002.

Per-ethylated pillar[6]arene

6.7

Chem. Soc. Rev. 2017, 46, 2479–2496.

Cucurbit[6]uril

3.9

Angew. Chem. Int. Ed. 2005, 44, 4844.

Cucurbit[7]uril

5.4

Chem. Soc. Rev. 2015, 44, 8747–8761.

Cucurbit[8]uril

6.9

Chem. Rev. 2016, 116, 12651–12652.

Table 2. Chemical structures and diameters of typical biphenarenes.

Macrocyclic host

Chemical structure

Diameter (Å)

Ref.

Terphen[3]arene

9.86

Angew. Chem. Int. Ed. 2019, 58, 3885.

Terphen[4]arene

13.85

Angew. Chem. Int. Ed. 2019, 58, 3885.

Terphen[5]arene

16.47

Acc. Chem. Res. 2022, 55, 916−929.

Terphen[6]arene

21.59

Acc. Chem. Res. 2022, 55, 916−929.

Quaterphen[3]arene

15.76

Angew. Chem. Int. Ed. 2019, 58, 3885.

Quaterphen[4]arene

17.86

Angew. Chem. Int. Ed. 2021, 60, 11288–11293

Quaterphen[5]arene

21.48

Angew. Chem. Int. Ed. 2019, 58, 3885.

Quaterphen[6]arene

30.82

Chem. Soc. Rev. 2020, 49, 1517–1544.

  1. Finally, since the authors repeatedly refer to the effects of ring size on the conformational behavior of traditional supramolecular hosts, they must clearly explain the conformational behavior of biphenarenes. What is known about the conformations of these compounds and how does conformation depend on ring size. Readers interested in using biphenarenes for their purposes must know more about these aspects, without which it is impossible to decide whether biphenarenes are alternatives to other macrocyclic hosts in certain areas.

Thank you very much for your kind comments. Biphenarenes with different number of structural units show different conformational behaviors. As shown in Figure S1, per-ethylated biphen[3]arene exhibits a distorted triangular-prism structure with no effective cavity in the solid state. Per-ethylated biphen[4]arene has a cuboid-like structure and exists in a ‘partial chair’ topology. It is also interesting to note that the biphenyl units in biphen[3,4]arenes can exist in two conformations, cis- and trans-conformations, according to the relative position of the two methylene linkers (Chem. Sci. 2015, 6, 197).

Figure S1. Crystal structures of per-ethylated biphen[3]arene (A and B) and per-ethylated biphen[4]arene (C and D).

  1. Another aspect that can be improved relates to the figures. At the moment, the authors almost only use figures from previous publications. These figures were probably useful in the corresponding publication to illustrate the reported results, but they are rather confusing outside of the original contexts. Figure 4 is an especially problematic example. Is it really necessary, for example, to include the NOESY NMR spectrum? Importantly, since the structures of biphenarenes investigated in the cited publications are all drawn differently, it is very difficult for the reader to easily understand the structural relationship of the compounds.

Many thanks for your kind comments and advices. All Figures have been carefully checked and revised. The NOESY NMR spectrum in Figure 4 has been removed.

  1. The authors consistently use the term "supramolecular macrocycle(s)", which is not well chosen. The macrocycles to which they refer are molecules prepared by typical concepts of molecular chemistry, and it is only their ability to act as receptors that qualifies them for supramolecular chemistry. The term "supramolecular" refers to properties or concepts but cannot be used to characterize covalently assembled molecules. This reviewer therefore strongly suggests to replace the term "supramolecular macrocycles" wherever it appears in the manuscript with terms such as "supramolecular hosts", "macrocyclic hosts", "macrocyclic receptors", "cyclophane-based hosts" etc.

Thank you very much for your kind advices. These were all done. We have changed the term “supramolecular macrocycles” to “macrocyclic hosts”.

  1. In the first sentence the authors write "...laid the foundation for the development of non-covalent interactions between molecules" which is awkward and wrong. Non-covalent interactions between molecules have always existed and they did not have to be developed. Pedersen's discovery "laid the foundation for the development of synthetic molecules that could engage in non-covalent interactions."

Thank you very much. This was done.

  1. Line 39: "the selective complexation of biphenarenes with cationic, anionic and neutral guest molecules" must read "the selective complexation of cationic, anionic and neutral guest molecules with biphenarenes."

Thank you very much. This was done.

  1. Line 61: "guest or molecules" should read "guest molecules" (a guest is usually also a molecule).

Thank you very much. This was done.

  1. Line 75: In the sentence "prone to deformation and folding due to the directivity of covalent bonds", the second part "due to the directivity (directionality is probably the better term) of covalent bonds" can be omitted. Importantly, this argument should also hold for biphenarenes. The authors must explain why "deformation and folding" is apparently irrelevant for these cyclophanes. It is also unclear what the word "it" in the next sentence refers to.

Many thanks for your kind comments and advices. These were all done. The argument of the directivity of covalent bonds also holds for biphenarenes. However, the structural units of biphenarenes are long and rigid. Therefore, there is no obvious deformation and folding of biphenarenes. For example, it is easy to obtain quaterphen[6]arenes with large cavity sizes (more than 3.0 nm) by selecting the long and rigid structural unit (Figure S2). In addition, “it” means “traditional macrocycle”.

Figure S2. Chemical and optimized structures of quaterphen[6]arene.

  1. Line 87: See comment referring to line 61.

Thanks very much. This was done.

  1. Line 115: Is it really correct to state that "Molecular recognition plays an important role in biological system[s], ion detection and environmental pollution control" only? Molecular recognition is relevant in so many areas that it is incorrect to name only three.

Thank you very much. This was done. We changed “Molecular recognition plays an important role in biological system, ion detection and environmental pollution control” to “Molecular recognition plays an important role in biological systems, ion detection, environmental pollution control, etc.”

  1. Line 118: Remove the word "topological", which in the context of this sentence has the same meaning as structure. "Topological structure" is therefore a pleonasm.

Thank you very much. This was done.

  1. Line 134: "protons of G2 exhibited field displacement" should read "protons of G2 were shielded."

Thank you for your advice. This was done.

  1. Line 163: The sentence "This is beneficial for applications in sensors, nanomaterials, ion or molecular transport, and supramolecular amphiphiles" could be improved by stating "This renders them useful as sensors, nanomaterials, ion or molecular transporters, and supramolecular amphiphiles."

Thank you for your advice. This was done.

  1. Line 171: "They can efficiently extract..." should read "They can be used to efficiently extract..."

Thank you for your advice. This was done.

  1. Line 212: The phrase "stably present in the cavity" indicates that benzene and toluene are unstable outside the cavity, which is wrong. Therefore "stably present" should probably better read "efficiently bound."

Thank you for your advice. This was done.

  1. Line 221: What is a "cyclic experiment"? Would it be better to state "Performing the experiment repeatedly"?

Thank you for your advice. This was done.

  1. Line 250: Again "cyclic adsorption properties" is not a well-chosen term. The "properties" are not "cyclic". Please rephrase.

Thank you for your advice. This was done. We changed “cyclic adsorption properties” to “high recyclability”.

  1. Line 313: The word "reach" in this sentence is likely incorrect. Would it be correct to say "these results indicate"?

Thank you for your advice. This was done.

  1. Line 370: This paragraph only repeats what has been written before. It can completely be removed except for the final sentence stating "Overall, the fascinating..."

Thank you for your advice. This was done.

Reviewer 2 Report

In this review article, Zhou et al. describe about a new macrocyclic host, biphenarenes, in terms of the advantages of the synthesis and host structures, molecular recognition and separation abilities, drug delivery and fluorescence sensing. The concept of "customizable cavity" seems to be very unique to the biphenarenes, and several functions are also explained based on this concept. In addition, as far as I checked, no review articles focusing on biphenarenes has yet been reported, and I think this review is significant in that regard. Therefore, I believe this article deserves to be accepted as a review with following minor modifications.

1) As described above, as far as I checked this is the first review article focusing on biphenarenes. But if there are previous related reviews, a brief mention of the comparison with it would be helpful.

2) The definition of biphenarenes is a bit vague to me. It would be good to briefly comment on the definition of the compounds, such as what structural features are called biphenarenes. This is because similar compounds and derivatives are discussed later, but their relationship to biphenarenes is sometimes difficult to understand.

3) The larger cavity of biphenarenes is emphasized based on the comparison with classical macrocycles. On the other hand, recently-developed macrocyclic compounds such as cycloparaphenylenes can also have large and rigid cavities, which allows them to bind large organic guests efficiently. Therefore, it may be better to cite them with a brief comment.

4) Figure 3 shows biphenarenes with different unit numbers (3 and 4). It would be helpful if the authors could briefly explain how the unit number is controlled in their synthesis.

5) In the section of molecular recognition, the excellent host ability of biphenarenes is emphasized, but I would like to see it quantitatively demonstrated by showing equilibrium constants in one of the cases. Without that, the excellence of biphenarenes as a host may not be conveyed.

6) I believe one of the pioneering example of nonporous adaptive crystals (NACs) is, to my knowledge, a paper reported by Atwood et al. (Science, 2002, 298, 1000). If I understand correctly, it would be better to cite it (or a better one) as the pioneering work that first proposed this concept.

7) Figure 7 and related parts show the recognition of a peptide by biphenarenes. It would be better to show the details of the structure and interaction modes of the peptide for helping to understand their host-guest chemistry.

8) Likewise, if possible, please provide details of the structure of LyeTxI in Figure 8, because the host-guest chemistry of them showing the strong binding constant is unique and interesting.

9) Some abbreviations may not be fully explained, especially on pages 12-13. Please check these thoroughly in this manuscript.

Author Response

Reply to reviewer 2:

  1. In this review article, Zhou et al. describe about a new macrocyclic host, biphenarenes, in terms of the advantages of the synthesis and host structures, molecular recognition and separation abilities, drug delivery and fluorescence sensing. The concept of "customizable cavity" seems to be very unique to the biphenarenes, and several functions are also explained based on this concept. In addition, as far as I checked, no review articles focusing on biphenarenes has yet been reported, and I think this review is significant in that regard. Therefore, I believe this article deserves to be accepted as a review with following minor modifications. As described above, as far as I checked this is the first review article focusing on biphenarenes. But if there are previous related reviews, a brief mention of the comparison with it would be helpful.

Many thank you for your kind comments and advices. This was done. A brief comment was made on previous reviews. The specific content is “So far, there are few reviews on biphenarenes, mainly focusing on the synthesis and structure of biphenarenes. In this review, structures and molecular recognition properties of biphenarenes are discussed in detail. In addition, applications of biphenarenes in adsorptive separation, drug delivery and fluorescence sensing are summarized”.

  1. The definition of biphenarenes is a bit vague to me. It would be good to briefly comment on the definition of the compounds, such as what structural features are called biphenarenes. This is because similar compounds and derivatives are discussed later, but their relationship to biphenarenes is sometimes difficult to understand.

Thank you for your kind comments and advices. This was done. Biphen[n]arenes, including basic biphen[n]arenes, functional biphen[n]arenes, and cage compounds, are new macrocyclic hosts appeared in the supramolecular world recently. Typically, biphenarenes are made up of 4,4’-biphenol or 4,4’-biphenol ether units linked by methylene bridges at the 3- and 3’- positions.

  1. The larger cavity of biphenarenes is emphasized based on the comparison with classical macrocycles. On the other hand, recently-developed macrocyclic compounds such as cycloparaphenylenes can also have large and rigid cavities, which allows them to bind large organic guests efficiently. Therefore, it may be better to cite them with a brief comment.

Thank you for your advice. This was done. A brief comment on cycloparaphenylenes was made. Relevant articles were cited as Ref. 59 and Ref. 60.

  1. Figure 3 shows biphenarenes with different unit numbers (3 and 4). It would be helpful if the authors could briefly explain how the unit number is controlled in their synthesis.

Thank you for your comment. This was done. A brief description about the synthesis of hydroxylated biphen[3]arene (H2) and hydroxylated biphen[4]arene (H3) was added. The number and position of substituents in structural units have an effect on the unit number of biphenarenes. For example, 2,2’, 3,3’, 4,4’-hexamethoxybiphenyl structural units produce biphen[3]arenes. However, 2,2’, 4,4’, 6,6’-hexamethoxyterphenyl structural units produce biphen[3]arenes and biphen[4]arenes. Furthermore, thin layer chromatography and gel permeation chromatography can be used to monitor the structure of biphenarenes during synthesis.

  1. In the section of molecular recognition, the excellent host ability of biphenarenes is emphasized, but I would like to see it quantitatively demonstrated by showing equilibrium constants in one of the cases. Without that, the excellence of biphenarenes as a host may not be conveyed.

Thank you for your advice. This was done. Association constants (Ka) of H1-H3 with different guests are showed in Table 1 of Section 3.

Table 1. Association constants (Ka) for the host−guest complexes of biphenarenes with different guests.

  1. I believe one of the pioneering examples of nonporous adaptive crystals (NACs) is, to my knowledge, a paper reported by Atwood et al. (Science, 2002, 298, 1000). If I understand correctly, it would be better to cite it (or a better one) as the pioneering work that first proposed this concept.

Thank you for your advice. This was done. The relevant article was cited as Ref. 120.

  1. Figure 7 and related parts show the recognition of a peptide by biphenarenes. It would be better to show the details of the structure and interaction modes of the peptide for helping to understand their host-guest chemistry.

Thank you for your advice. This was done. The structure and interaction modes of the peptide have been added in Figure 7.

  1. Likewise, if possible, please provide details of the structure of LyeTxI in Figure 8, because the host-guest chemistry of them showing the strong binding constant is unique and interesting.

Thank you for your advice. This was done. The structure of LyeTxI has been added in Figure 8.

  1. Some abbreviations may not be fully explained, especially on pages 12-13. Please check these thoroughly in this manuscript.

Thank you for your advice. These were all done. We have checked and revised these thoroughly in this manuscript.

Round 2

Reviewer 1 Report

The authors adequately responded to my previous comments. Accordingly, the quality of the manuscript sufficiently improved and acceptance can now be recommended. One final suggestion for improvement: The paragraph on page 4:

"Large cavity. Traditional macrocycles usually regulate the cavity size by increasing the number of structural units. However, the construction of macrocyclic hosts by increasing the number of structural units cannot obtain effective cavities because of structural distortion and folding. However, the structures of traditional macrocycles are prone to deformation and folding due to the directivity of covalent bonds. As a result, it cannot form effective cavities. The synthesis of biphenarenes is based on the linking of reaction modules to form macrocycles by Friedel-Crafts alkylation. The cavity sizes of biphenarenes can be increased through prolonging the functional modules rather than increasing the number of structural units."

can potentially be improved by rephrasing and changing the sequence of the arguments in the following way:

"Large cavity. Like many other structurally related macrocyclic hosts, biphenarenes are formed from suitable electron-rich aromatic building blocks and formaldehyde by repeated Friedel-Crafts alkylations. The normal strategy to increase cavity size involves increasing the number of subunits along the ring, but this can concomitantly lead to an increase of conformational flexibility accompanied by a collapse of the cavity. In contrast, the cavity size of biphenarenes is increased by incorporating spacers (or functional modules) between the terminal aromatic units of the building blocks, making macrocycles with large cavity easily accessible."

Minor improvements still required.

Author Response

The authors adequately responded to my previous comments. Accordingly, the quality of the manuscript sufficiently improved and acceptance can now be recommended. One final suggestion for improvement: The paragraph on page 4:

“Large cavity. Traditional macrocycles usually regulate the cavity size by increasing the number of structural units. However, the construction of macrocyclic hosts by increasing the number of structural units cannot obtain effective cavities because of structural distortion and folding. However, the structures of traditional macrocycles are prone to deformation and folding due to the directivity of covalent bonds. As a result, it cannot form effective cavities. The synthesis of biphenarenes is based on the linking of reaction modules to form macrocycles by Friedel-Crafts alkylation. The cavity sizes of biphenarenes can be increased through prolonging the functional modules rather than increasing the number of structural units.” can potentially be improved by rephrasing and changing the sequence of the arguments in the following way:

“Large cavity. Like many other structurally related macrocyclic hosts, biphenarenes are formed from suitable electron-rich aromatic building blocks and formaldehyde by repeated Friedel-Crafts alkylations. The normal strategy to increase cavity size involves increasing the number of subunits along the ring, but this can concomitantly lead to an increase of conformational flexibility accompanied by a collapse of the cavity. In contrast, the cavity size of biphenarenes is increased by incorporating spacers (or functional modules) between the terminal aromatic units of the building blocks, making macrocycles with large cavity easily accessible.”

Many thank you for your kind advice and comment. This was done.
